# Quality of reporting of randomized controlled trials in polycystic ovary syndrome

**DOI:** 10.1186/1745-6215-10-106

**Published:** 2009-11-20

**Authors:** Anna Partsinevelou, Elias Zintzaras

**Affiliations:** 1 Department of Biomathematics, University of Thessaly School of Medicine, Larissa, Greece; 2 Center for Clinical Evidence Synthesis, The Institute for Clinical Research and Health Policy Studies, Tufts Medical Center, Tufts University School of Medicine, Boston, USA

## Abstract

**Background:**

Inadequate reporting of randomized controlled trials (RCTs) is associated with biased estimates of treatment effects. The reporting quality of RCTs involving patients with polycystic ovary syndrome (PCOS) is unknown. The purpose of this study was to assess the reporting quality of RCTs involving patients with PCOS using a standardized tool based on the Consolidated Standards of Reporting Trials (CONSORT) statement.

**Methods:**

We searched PubMed database for English-language RCTs involving patients with PCOS. Quality of reporting was assessed using a 24-item questionnaire based on the revised CONSORT checklist. Reporting was evaluated overall, and for pre- and post-CONSORT periods. RCTs on PCOS associated with fertility and non-fertility disturbances were also evaluated separately.

**Results:**

Nine of the 24 items were reported in less than 50% of the studies, while a significant improvement (P < 0.05) was detected in 12 of 24 items (50%) over the two CONSORT periods. The RCTs on PCOS with reference to fertility seem to have adhered better to CONSORT statement than RCTs not associated to fertility.

**Conclusion:**

There is empirical evidence of suboptimal reporting quality of RCTs in PCOS. Endorsement of the CONSORT statement may optimize the reporting quality and enhance the validity of research.

## Background

Randomized controlled trials (RCTs) are considered the best tool for establishing effectiveness due to minimization of bias in evaluating new treatment strategies [1-3]. RCTs represent a key research activity with the potential to improve the quality of health care and control costs through careful comparison of alternative treatments [4,5]. However, the recent flood of available information in biomedical journals during the last years has raised problems in a variety of areas, such as publication or selection bias and retraction of invalid literature [1,6,7].

The assessment of the methodological quality of a trial is integrally linked with the quality of reporting; that is, the extent to which a report provides information about the design, conduct, and analysis of the trial [8]. Reports often omit important methodological details. For example, only 9% of 206 RCTs published in obstetrics and gynaecology journals described both sequence generation and allocation concealment [9]. Inadequate reporting and design are associated with biased estimates of treatment effects. The bias associated with defects in the conduct of RCTs varies with the type of outcome. In trials with subjectively assessed outcomes, lack of adequate allocation concealment or blinding tends to produce over-optimistic estimates of the effect of interventions [10]. In general, faulty reporting reflects faulty methods and a well-conducted but badly-reported trial will be misclassified [8,11,12]. Previous studies [11,13,14] indicate that reports of low-quality RCTs, compared with reports of higher-quality ones, overestimate the effectiveness of interventions by about 30% across a variety of health care conditions.

In response to concerns about quality of reporting of RCTs, an international group of scientists and editors developed and published in 1996, a common checklist for items to include in reports of RCTs, known as the Consolidated Standards of Reporting Trials (CONSORT) statement [3,15]. In 2001 the original CONSORT statement was revised and updated to its current version, consisting of a 22-item checklist and a four-stage flow diagram, in order to facilitate critical appraisal and interpretation of RCTs by providing guidance to authors about how to improve the reporting of their trials [16-21]. Although the content of the revised checklist is similar to that of the original one, some previously combined items are separated in the new version. An important addition to the checklist is the reporting of the intention to treat (ITT) analysis [18]. Since its publication in 1996, the CONSORT statement has been widely supported [19], has been translated into several languages and has an Internet presence http://www.consort-statement.org to enhance awareness and dissemination [17]. Its use is recommended by the International Committee of Medical Journal Editors, the Council of Science Editors, and the World Association of Medical Editors [17]; to date, more than 300 biomedical journals, have adopted these recommendations [4,14-18].

There has been so far no published systematic evaluation on the quality of RCTs involving patients with polycystic ovary syndrome (PCOS) based on the adherence to the CONSORT statement. Given that the reporting of fundamental methodological details is critical to interpret the results of RCTs and the assessment of the quality of the medical literature is essential, this topic was considered sufficiently important to merit study. This particular syndrome was chosen due to its prevalence on women of reproductive age and its impact on the reproductive and cardiovascular system, necessitating a specific and tailored treatment plan. This gynaecological disturbance, which is diagnosed in 6 to 7% of women [22], is a heterogeneous endocrine disorder of uncertain aetiology and a common cause of anovulatory infertility, menstrual dysfunction, and hirsutism [22,23]. Aim of this study was to determine the overall reporting quality of published RCTs on PCOS using a 24-item questionnaire based on the revised CONSORT checklist [17,18].

## Methods

### Studies Selection and Data Extraction

Literature for this review was systematically identified by searching PubMed for papers on RCTs involving patients with PCOS, published between January 1, 1990 and February 29, 2008. The selection of this starting date was made in order to avoid confounding factors such as editorial restrictions and unavailability of electronic publishing, which apply in publications, mainly, before 1990, and to focus on the literature of RCTs that has been informative for clinical decision-making in the field over the last two decades. We used as filters the "Randomized Controlled Trial" type of article, "English" language, "Humans", "Female" gender, and as a search criterion the following term: "polycystic ovary syndrome". Trials were eligible if they had randomly assigned participants to at least two treatment arms and included patients with PCOS. All references cited in the retrieved articles were also reviewed to identify additional published work not indexed by PubMed. Articles were independently screened for eligibility by the two authors, who were blinded to each other's responses. Any discrepancies were resolved through consensus and reference to the abstracts or articles.

The revised CONSORT checklist includes a 22-item-questionaire. However, in an attempt to determine better internal and external validity, two items from the CONSORT checklist, namely the reporting of recruitment and follow-up periods and the reporting of outcomes, were divided each of them into two subcategories (recruitment and follow-up, reporting of outcomes and precision of their estimated effect). Hence, based on CONSORT reporting items, we developed a 24-items data extraction sheet (Table 1). All items were investigated in terms of whether they were reported, not whether they were actually carried out during the trial. Alternatives responses (apart from yes or no) and unclear responses to each question were coded as negative responses.

**Table 1:** Proportion of reporting of 24 data items in a total of 264 randomized clinical trials in polycystic ovary syndrome by publication period (pre- and post-CONSORT and combined)*

Data items	Combined 1990-2008 (n = 264)†	Pre-CONSORT 1990-1995 (n = 27)	Post-CONSORT 1996-2008 (n = 237)	Odds Ratio and 95% confidence intervals^¥^	P-value‡
***TITLE/ABSTRACT***					

**1. Randomized in title/abstract**	0.84 (222)	0.78 (21)	0.85 (201)	1.60(0.60, 4.23)	0.4

***INTRODUCTION***					

**2. Scientific background in introduction**	0.89 (234)	0.89 (24)	0.89 (210)	0.97(0.27, 3.45)	0.99

***METHODS***					

**3. Eligibility criteria for participants**	0.67(176)	0.19 (5)	0.72 (171)	11.40(4.14, 31.35)	<0.01

**4. Precise details of the interventions in each arm**	0.99 (261)	1.00 (27)	0.99 (234)	1.22(0.06, 24.21)	0.99

**5. Objectives**	0.96 (254)	0.85 (23)	0.97 (231)	6.70(1.76, 25.46)	0.01

**6. End-points**	0.65 (171)	0.22 (6)	0.7 (165)	8.02(3.11, 20.71)	<0.01

**7. Sample size**	0.30 (80)	0.15 (4)	0.32 (76)	2.71(0.91, 8.12)	0.08

**8. Method of randomization (sequence generation)**	0.56 (148)	0.30 (8)	0.59 (140)	3.43(1.44, 8.15)	<0.01

**9. Allocation concealment**	0.39 (104)	0.07 (2)	0.43 (102)	9.44(2.19, 40.49)	<0.01

**10. Implementation of randomization**	0.21 (56)	0.00 (0)	0.24 (56)	17.12(1.03, 285.19)	<0.01

**11. Blinding (masking)**	0.45 (120)	0.37 (10)	0.46 (110)	1.47(0.65, 3.35)	0.42

**12. Statistical methods**	0.95 (252)	0.89 (24)	0.96 (228)	3.17(0.80, 12.50)	0.11

***RESULTS***					

**13. Participant flow**	0.625 (165)	0.22 (6)	0.67 (159)	7.13(2.77, 18.39)	<0.01

**14. Periods: a. Recruitment**	0.37 (98)	0.04 (1)	0.41 (97)	18.01(2.40, 134.99)	<0.01

**b. Follow-up**	0.32 (85)	0.04 (1)	0.35 (84)	14.27(1.90, 107.07)	<0.01

**15. Baseline data**	0.80 (212)	0.52 (14)	0.84 (198)	4.71(2.06, 10.80)	<0.01

**16. "Intention-to-treat" analysis**	0.125 (33)	0.00 (0)	0.14 (33)	9.01(0.54, 151.26)	0.03

**17. a. Outcomes and**	0.99 (262)	1.00 (27)	0.99 (235)	1.71(0.08, 36.60)	0.99

**b. Estimation of effects**	0.54 (143)	0.44 (12)	0.55 (131)	1.54(0.69, 3.44)	0.31

**18. Ancillary analyses**	0.39 (103)	0.15 (4)	0.42 (99)	4.13(1.38, 12.30)	0.01

**19. Adverse events**	0.50 (132)	0.33 (9)	0.52 (123)	2.16(0.93, 5.00)	0.1

***DISCUSSION***					

**20. Interpretation of the results**	0.98 (260)	1.00 (27)	0.98 (233)	0.94(0.05, 18.00)	0.99

**21. Generalizability**	0.92 (243)	0.89 (24)	0.92 (219)	1.52(0.42, 5.54)	0.46

**22. Overall evidence**	0.90 (237)	0.81 (22)	0.91 (215)	2.22(0.77, 6.45)	0.17

* CONSORT = Consolidated Standards of Reporting Trials.

† The percentage of articles reporting the CONSORT item.

^¥ ^Odds ratio of reporting an item at post-CONSORT period relative to pre-CONSORT.

‡ *P*-values from Fisher's exact test for testing the association between reporting an item and publication period.

### Statistical Methods

The articles were grouped in two publication periods, i.e. 1990-1995 (pre-CONSORT), and 1996-2008 (post-CONSORT), whereon reporting was assessed. In addition, we compared the adherence to the CONSORT statement in published reports of RCTs on PCOS with reference to fertility with the remaining eligible papers concerning non-fertility issues on PCOS.

Although all items in the CONSORT checklist are considered important as to improve the quality of reports of RCTs, emphasis was placed on reporting of methodological items which are more objective, that is sample size, method of randomization and allocation concealment, performed statistical methods, description of baseline data, precision of estimated effect size and reporting of ITT analysis. Reporting of results according to the intention-to-treat principle was analyzed in more details because deviations from this principle can lead to over-optimistic and biased results [24]. The ITT analysis includes all randomized patients in the groups to which they were randomly assigned, regardless of their compliance with the entry criteria, the treatment they actually received, and subsequent withdrawal from treatment or deviation from the protocol [24,25].

Comparisons between pre- and post-CONSORT periods were performed by calculating the odds ratio (OR), and the respective 95% confidence interval, of reporting an item at post-CONSORT period relative to pre-CONSORT. Also, the association between reporting an item and publication period was tested using the Fisher' exact test. The cutoff point for statistical significance was set at the two-sided 0.05 level.

## Results

**Figure 1:**
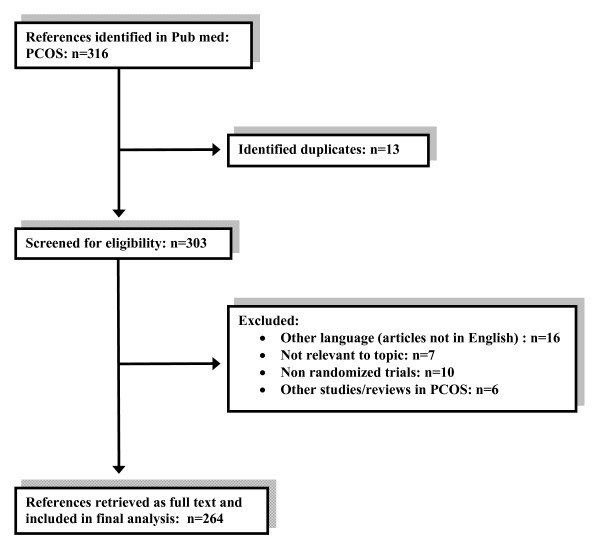
**Flow diagram of citations through the retrieval and the screening process**. RCT = randomized controlled trial; PCOS = Polycystic Ovary Syndrome.

Our search strategy identified 316 studies, of which 264 met the inclusion criteria (Fig. 1). A full list of the 264 reports that were retrieved as full-text and included in the final analysis is found in http://biomath.med.uth.gr. Of these articles, 27 were published in the pre-CONSORT period (1990-1995) and 237 in the post-CONSORT period (1996-2008). The average annual publication rate of RCTs in PCOS increased from 5.5 publications per year for the period 1990-1995 to 20.53 for the period 1996-2008, representing a mean increase of approximately fifteen publications each year over the period under investigation. The articles were retrieved from 57 journals of which 45 have not endorsed the CONSORT statement (Table 2). The majority of papers (59.1%) were published in journals that are non-endorsers, including some of wide circulation, such as Fertility and Sterility.

**Table 2:** Distribution of papers by journals

Journal	Papers*	Consort endorser†
Fertility and sterility	24.62%	no

Human Reproduction	17.80%	yes

The Journal of clinical endocrinology and metabolism	17.42%	yes

Gynecological endocrinology	4.17%	no

Clinical endocrinology (Oxf)	2.65%	no

The Journal of reproductive medicine	2.27%	no

European journal of endocrinology	1.89%	no

International Journal of Gynaecology Obstetrics	1.89%	no

Others (39 journals)	21.59%	no

Others (10 journals)	5.68%	yes

*The percentage of articles published in the journal

†According to the list "CONSORT Endorsers - Journals" provided in http://www.consort-statement.org.

The consistency between the two authors for assessment of all CONSORT items was determined using Cohen's kappa statistics. This method involves the degree of reviewers' agreement on whether an item was reported or not. For calculation of κ, all items were considered together for each paper and a good agreement between the two reviewers was found with κ = 0.92 (0.88-0.96) in general [26].

When the 264 RCTs are considered together, 9 of the 24 items were reported in less than 50% of the studies, such as sample size, allocation concealment, implementation of randomization and blinding (Table 1). Most reports, 231 of 264 (87.5%) did not give any information about "intention-to-treat" analysis and those that report it were conducted after the implementation of the CONSORT statement. Similarly, implementation of randomization was reported only in 21% (56/264) of the studies, all published after 1995. Items that were reported in almost 90% of the studies were the details of the interventions in each group, the hypothesis and objectives of each study, the statistical methods, the description of outcomes and the summary results (interpretation, generalizability and overall evidence).

After comparison of the two periods, a significant improvement (P < 0.05) was detected in 12 of 24 items (50%). These include reporting of eligibility criteria for participants and locations where the data were collected, hypothesis and objectives, primary and secondary outcome measures, method of randomization (sequence generation and allocation concealment), implementation of randomization, participant flow, periods of recruitment and follow-up, baseline data, "intention-to-treat" analysis and ancillary analyses. The aforementioned items, except "intention-to-treat" analysis, are much more likely to be reported in the post-CONSORT period than in the pre-CONSORT, (see respective ORs at Table 1). An overall improvement in reporting across all items was detected after CONSORT statement's implementation, except for the reporting of the precise details of the interventions, the outcomes and the interpretation of the results that showed a minor, not statistically significant reduction.

Among the 264 RCTs, 122 were associated with fertility and 142 with non-fertility disturbances. The proportion of reporting of the 24 items in these categorized trials is displayed at Table 3 and 4. In both of them, approximately 7 of the 24 items were identified in more than 90% of the studies. More specifically, 9 of the 24 items were reported in less than 50% of the RCTs associated with fertility, while similarly 8 of the 24 items were detected in less than 50% of the RCTs of the other group. In both tables the items that showed the greatest improvement were those that were under-reported pre-CONSORT, such as allocation concealment with OR = 20.52 (2.62, 160.49) and method of randomization with OR = 3.67 (1.25, 10.74). The reporting in papers related to fertility in the pre-CONSORT period was worse than those relevant to non-fertility issues. So, the RCTs on PCOS with reference to fertility seem to have adhered better to CONSORT statement, with significant improvement (P < 0.05) in 13 of the 24 items, in contrast with the RCTs not associated to fertility, which showed significant improvement in only 6 of the 24 items.

**Table 3:** Proportion of reporting of 24 data items in 122 randomized clinical trials in polycystic ovary syndrome associated with fertility by publication period (pre- and post-CONSORT and combined)*

Data items	Combined 1990-2008 (n = 122) †	Pre-CONSORT 1990-1995 (n = 17)	Post-CONSORT 1996-2008 (n = 105)	Odds Ratio and 95% confidence intervals^¥^	P-value‡
***TITLE/ABSTRACT***					

**1. Randomized in title/abstract**	0.89(108)	0.76(13)	0.90(95)	2.92(0.80, 10.69)	0.11

***INTRODUCTION***					

**2. Scientific background in introduction**	0.89(108)	0.94(16)	0.88(92)	0.44(0.05, 3.62)	0.69

***METHODS***					

**3. Eligibility criteria for participants**	0.62(76)	0.12(2)	0.70(74)	17.90(3.86, 83.00)	<0.01

**4. Precise details of the interventions in each arm**	0.98(120)	1.00(17)	0.98(103)	1.18(0.05, 25.70)	0.99

**5. Objectives**	0.97(118)	0.82(14)	0.99(104)	22.29(2.17, 229.28)	0.01

**6. End-points**	0.63(77)	0.18(3)	0.70(74)	11.14(2.99, 41.52)	<0.01

**7. Sample size**	0.38(46)	0.12(2)	0.42(44)	5.41(1.18, 24.87)	0.03

**8. Method of randomization (sequence generation)**	0.62(76)	0.35(6)	0.67(70)	3.67(1.25, 10.74)	0.02

**9. Allocation concealment**	0.49(60)	0.06(1)	0.56(59)	20.52(2.62, 160.49)	<0.01

**10. Implementation of randomization**	0.23(28)	0.00(0)	0.27(28)	12.87(0.75, 221.13)	0.01

**11. Blinding (masking)**	0.41(50)	0.47(8)	0.40(42)	0.75(0.27, 2.10)	0.61

**12. Statistical methods**	0.95(116)	0.88(15)	0.96(101)	3.37(0.57, 20.00)	0.19

***RESULTS***					

**13. Participant flow**	0.60(73)	0.35(6)	0.64(67)	3.23(1.11, 9.44)	0.03

**14. Periods: a. Recruitment**	0.51(62)	0.06(1)	0.58(61)	22.18(2.84, 173.55)	<0.01

**b. Follow-up**	0.26(32)	0.00(0)	0.30(32)	15.48(0.90, 265.24)	0.01

**15. Baseline data**	0.73(89)	0.47(8)	0.77(81)	3.80(1.32, 10.91)	0.02

**16. "Intention-to-treat" analysis**	0.15(18)	0.00(0)	0.17(18)	7.40(0.43, 128.64)	0.07

**17. a. Outcomes and**	0.99(121)	1.00(17)	0.99(104)	1.99(0.08, 50.85)	0.09

**b. Estimation of effects**	0.45(55)	0.41(7)	0.46(48)	1.20(0.43, 3.40)	0.79

**18. Ancillary analyses**	0.43(52)	0.12(2)	0.48(50)	6.82(1.48, 31.31)	0.01

**19. Adverse events**	0.49(60)	0.24(4)	0.53(56)	3.71(1.14, 12.14)	0.04

***DISCUSSION***					

**20. Interpretation of the results**	0.98(120)	1.00(17)	0.98(103)	1.18(0.05, 25.70)	0.99

**21. Generalizability**	0.93(113)	0.94(16)	0.92(97)	0.76(0.09, 6.47)	0.99

**22. Overall evidence**	0.93(113)	0.94(16)	0.92(97)	0.76(0.09, 6.47)	0.99

* CONSORT = Consolidated Standards of Reporting Trials.

† The percentage of articles reporting the CONSORT item

* CONSORT = Consolidated Standards of Reporting Trials.

† The percentage of articles reporting the CONSORT item.

^¥ ^Odds ratio of reporting an item at post-CONSORT period relative to pre-CONSORT.

‡ *P*-values from Fisher's exact test for testing the association between reporting an item and publication period.

**Table 4:** Proportion of reporting of 24 data items in 142 randomized clinical trials in polycystic ovary syndrome associated with non-fertility disturbances by publication period (pre- and post-CONSORT and combined)*

Data items	Combined 1990-2008 (n = 142) †	Pre-CONSORT 1990-1995 (n = 10)	Post-CONSORT 1996-2008 (n = 132)	Odds Ratio and 95% confidence intervals^¥^	P-value‡
***TITLE/ABSTRACT***					

**1. Randomized in title/abstract**	0.80(114)	0.80(8)	0.80(106)	1.02(0.20, 5.09)	0.99

***INTRODUCTION***					

**2. Scientific background in introduction**	0.89(126)	0.80(8)	0.89(118)	2.11(0.41, 10.92)	0.31

***METHODS***					

**3. Eligibility criteria for participants**	0.70(100)	0.30(3)	0.73(97)	6.47(1.58, 26.40)	0.01

**4. Precise details of the interventions in each arm**	0.99(141)	1.00(10)	0.99(131)	4.17(0.16, 108.94)	0.99

**5. Objectives**	0.96(136)	0.90(9)	0.96(127)	2.82(0.30, 26.80)	0.36

**6. End-points**	0.66(94)	0.30(3)	0.69(91)	5.18(1.27, 21.04)	0.03

**7. Sample size**	0.24(34)	0.20(2)	0.24(32)	1.28(0.26, 6.34)	0.99

**8. Method of randomization (sequence generation)**	0.51(72)	0.20(2)	0.53(70)	4.52(0.92, 22.07)	0.05

**9. Allocation concealment**	0.31(44)	0.10(1)	0.33(43)	4.35(0.53, 35.43)	0.17

**10. Implementation of randomization**	0.20(28)	0.00(0)	0.21(28)	5.73(0.33, 100.73)	0.21

**11. Blinding (masking)**	0.49(70)	0.20(2)	0.52(68)	4.25(0.87, 20.77)	0.09

**12. Statistical methods**	0.96(136)	0.90(9)	0.96(127)	2.82(0.30, 26.80)	0.36

***RESULTS***					

**13. Participant flow**	0.65(92)	0.00(0)	0.70(92)	47.96(2.74, 838.36)	<0.01

**14. Periods: a. Recruitment**	0.25(36)	0.00(0)	0.27(36)	7.94(0.45, 139.05)	0.07

**b. Follow-up**	0.37(53)	0.10(1)	0.39(52)	5.85(0.72, 47.55)	0.09

**15. Baseline data**	0.87(123)	0.60(6)	0.89(117)	5.20(1.32, 20.56)	0.03

**16. "Intention-to-treat" analysis**	0.11(15)	0.00(0)	0.11(15)	2.77(0.15, 49.65)	0.61

**17. a. Outcomes and**	0.99(141)	1.00(10)	0.99(131)	4.17(0.16, 108.94)	0.99

**b. Estimation of effects**	0.62(88)	0.50(5)	0.63(83)	1.69(0.47, 6.15)	0.51

**18. Ancillary analyses**	0.36(51)	0.20(2)	0.37(49)	2.36(0.48, 11.57)	0.33

**19. Adverse events**	0.51(72)	0.50(5)	0.51(67)	1.03(0.28, 3.37)	0.99

***DISCUSSION***					

**20. Interpretation of the results**	0.99(140)	1.00(10)	0.98(130)	2.49(0.11, 55.22)	0.99

**21. Generalizability**	0.92(130)	0.80(8)	0.92(122)	3.05(0.57, 16.34)	0.21

**22. Overall evidence**	0.87(124)	0.60(6)	0.89(118)	5.62(1.41, 22.36)	0.02

* CONSORT = Consolidated Standards of Reporting Trials.

† The percentage of articles reporting the CONSORT item

^¥ ^Odds ratio of reporting an item at post-CONSORT period relative to pre-CONSORT.

‡ *P*-values from Fisher's exact test for testing the association between reporting an item and publication period.

## Discussion

There are several limitations to our study. We searched only in PubMed, which is the most common used medical database, for the eligible RCTs and did not extent to the Cochrane Collaboration database to combine our results with one more sensitive search strategy for RCTs. However, a more comprehensive literature search would be costly and time-consuming. In addition, trials which are difficultly retrieved tend to be of lower methodological quality and thus, bias could be introduced [27]. We considered only articles published in English, which could lead to language bias, since authors tend to publish RCTs in English-language journals if the results are statistically significant [28]; however, this risk of bias is limited given that only 5.7% of the articles captured by our search strategy were published in other languages [28]. Another limitation is that we did not assess the RCT methodological quality directly, as we did not verify the information from the authors or their protocols. Important methodological details of the trials may not be published and thus not evaluated. Devereaux et al. concluded through their observational study that authors of RCTs often use allocation concealment and blinding, despite the failure to report them [29]. The reporting of methodological aspects of RCTs does not necessarily reflect the conduct of the trial [30]. Additionally, although the CONSORT checklist was revised in 2001, we decided to use the time periods 1990-1995 and 1996-2008 because the effort of improving the quality of RCTs began in 1996 with the original CONSORT statement and the items of the original checklist still exist in the current version [17,18]. Thereupon, an imbalance occurred in the amount of articles compared in the two periods. Finally, there is not any reliable and valid tool to assess the methodological quality of RCTs, so our reporting quality scores are not verified. Many scales are used to evaluate the methodological quality of RCTs, but most of them did not follow methodological standards during development and have not been adequately tested for validity and reliability in the areas to which they have been applied [31]. Despite these limitations, our results have good internal validity, since we used an evaluation instrument and the selection and evaluation processes were independently performed by two reviewers. A substantial degree of agreement beyond chance for most criteria was achieved, lending internal validity to our results.

The motivation for this observational study was of statistical kind, thereby we compared two time periods, i.e. 1990-1995 and 1996-2008, in order to detect any improvements of reporting of CONSORT items, guiding on improvement of validity and quality of RCTs [4,14,20]. We concluded that the reporting quality of RCTs on patients with PCOS between 1990 and 2008 is suboptimal. Even though there was a statistically significant improvement over time in several items, such as participant flow and baseline data, the trials were limited in their reference of key methodologies; explicit report that "intention-to-treat" analysis was performed occurred only in 12.5% of the RCTs. Methods of randomization (allocation concealment and sequence generation), blinding, and analysis according to ITT principle are essential for internal validity so as to avoid selection, performance, detection and attrition bias [8]. In addition, the lack of adequate reporting of these key items has been associated with distortions in estimates of the treatment effect [11,13]. Our findings are in agreement with similar studies assessing the reporting quality of RCTs published in other medical subspecialties [4,9]. Lai et al. found that only 30% of the RCTs in the primary treatment of brain tumors reported allocation concealment, blinding and ITT [32]. Similarly, Dias et al. identified that 51% of the RCTs on subfertility provided details on the randomization method [33].

The suboptimal reporting quality of RCTs on patients with PCOS is also related with the fact that from the 264 papers included in the study only 40.9% came from journals that have endorsed the CONSORT statement. The majority of the journals in the field of obstetrics and gynecology are non-endorsers, including journals with relatively high impact factors, such as Fertility and Sterility. In an analysis of journals before and after the endorsement of CONSORT statement, it was found that the descriptions of the method of sequence generation, participant flow and total CONSORT items were better after endorsement of CONSORT (standardized mean difference, 3.67 items; 95% CI, 2.09-5.25) [16]. Thus, the suboptimal reporting quality is not merely an adherence but possibly an awareness issue of the CONSORT guidelines.

The intention of the CONSORT statement was to improve the quality of reporting of RCTs. Studies on the quality of reports of RCTs before and after the publication of CONSORT suggest that the adoption of this statement is associated with improved reporting of RCTs [14,16]. Health care providers depend on the reporting of methodological factors in the reports of RCTs to allow them to determine the validity of the trials upon which they base their clinical practice and their treatment guidelines [34,35]. To assess the strengths and limitations of RCTs, they need and deserve to know the quality of the methods being used [11]. With endorsement by more journals, and greater editorial efforts to ensure that improved authors' compliance, the CONSORT statement could begin to yield its intended benefits [15]. Consequently, reconsideration of editorial policies regarding enhanced adoption and adherence to the CONSORT statement is an issue that merits particular attention.

## Conclusion

The knowledge gained from this study should be viewed as an opportunity for improved adherence and increased awareness of the CONSORT statement. The present study provided empirical evidence of suboptimal reporting quality of RCTs in PCOS and highlights the need for endorsement of the CONSORT statement by journals in the field of gynecology and obstetrics, as well as the need for increased vigilance of authors and editors regarding compliance of manuscripts to the CONSORT statement.

## Competing interests

The authors declare that they have no competing interests.

## Authors' contributions

EZ conceived the study, assessed the articles and performed the statistical analysis. AP retrieved and assessed the articles. Both authors drafted, read and approved the manuscript.
